# Residue 315 regulates V3 exposure and V3 antibody recognition on HIV subtype B and C viruses

**DOI:** 10.1186/1742-4690-9-S2-P54

**Published:** 2012-09-13

**Authors:** S Manhas, B Clark, MK Gorny, S Zolla-Pazner, R Pantophlet

**Affiliations:** 1 Simon Fraser University, Burnaby, Canada; 2 New York University School of Medicine, New York, NY, USA

## Background

V3 mAbs often neutralize HIV subtype B viruses but exhibit poor neutralizing activity against subtype C viruses. This limited activity is typically attributed to masking of the V3 region on subtype C viruses. However, while relatively much effort has been devoted to exploring accessibility of the V3 region on subtype B viruses, V3 exposure and the mechanism(s) that might restrict V3 exposure on subtype C viruses has yet to be understood. Here we have focused on exploring the significance of the conserved V3 tip motifs GPGR and GPGQ of subtype B and C viruses for antibody recognition.

## Methods

Position 315 in representative subtype B (SS1196) and subtype C viruses (ZM249M, CAP45) was switched to Gln and Arg, respectively, to assess the effect of the conserved Arg/Gln at position 315 on V3-specific neutralization. Neutralization sensitivities of the parental and mutant viruses were assessed in a single-round pseudovirus neutralization assay using a panel of neutralizing V3-specific mAbs with varying fine specificities for the V3 tip.

## Results

Subtype B virus SS1196 was neutralized by all V3-specific mAbs tested here (B4e8, 2219, 268-D, 2557, 3074 and HGN194). In contrast, though as expected, mutant SS1196_R315Q was resistant to neutralization by mAbs B4e8 and 268-D, both of which require the Arg at position 315 for binding. Unexpectedly, the remaining V3 mAbs were also unable to neutralize SS1196_R315Q, despite not requiring an Arg residue at position 315 for binding. For the subtype C viruses the exact opposite was observed; both ZM249M and CAP45 were generally insensitive to antibody neutralization yet the Q315R mutants were strikingly sensitive.

## Conclusion

The results suggest that V3 may be more accessible to antibody than previously appreciated in at least some subtype C viruses. However, the data also suggest that a Gln at position 315 modulates exposure of V3. Further elucidation of this mechanism is underway.

